# Multifaceted role of redox pattern in the tumor immune microenvironment regarding autophagy and apoptosis

**DOI:** 10.1186/s12943-023-01831-w

**Published:** 2023-08-10

**Authors:** Yuqing Ren, Ruizhi Wang, Siyuan Weng, Hui Xu, Yuyuan Zhang, Shuang Chen, Shutong Liu, Yuhao Ba, Zhaokai Zhou, Peng Luo, Quan Cheng, Qin Dang, Zaoqu Liu, Xinwei Han

**Affiliations:** 1 Department of Interventional Radiology, The First Affiliated Hospital of Zhengzhou University, Zhengzhou, 450052 Henan China; 2 Department of Respiratory and Critical Care Medicine, The First Affiliated Hospital of Zhengzhou University, Zhengzhou, 450052 Henan China; 3 Center of Reproductive Medicine, The First Affiliated Hospital of Zhengzhou University, Zhengzhou, Henan 450052 China; 4 Department of Pediatric Urology, The First Affiliated Hospital of Zhengzhou University, Zhengzhou, 450052 Henan China; 5 Department of Oncology, Zhujiang Hospital, Southern Medical University, Guangzhou, Guangdong 510282 China; 6 Department of Neurosurgery, Xiangya Hospital, Central South University, Changsha, Hunan 410008 China; 7 Department of Colorectal Surgery, The First Affiliated Hospital of Zhengzhou University, Zhengzhou, 450052 Henan China

**Keywords:** Redox and ROS, Apoptosis, Autophagy, Immunity, Cancer therapy

## Abstract

The reversible oxidation-reduction homeostasis mechanism functions as a specific signal transduction system, eliciting related physiological responses. Disruptions to redox homeostasis can have negative consequences, including the potential for cancer development and progression, which are closely linked to a series of redox processes, such as adjustment of reactive oxygen species (ROS) levels and species, changes in antioxidant capacity, and differential effects of ROS on downstream cell fate and immune capacity. The tumor microenvironment (TME) exhibits a complex interplay between immunity and regulatory cell death, especially autophagy and apoptosis, which is crucially regulated by ROS. The present study aims to investigate the mechanism by which multi-source ROS affects apoptosis, autophagy, and the anti-tumor immune response in the TME and the mutual crosstalk between these three processes. Given the intricate role of ROS in controlling cell fate and immunity, we will further examine the relationship between traditional cancer therapy and ROS. It is worth noting that we will discuss some potential ROS-related treatment options for further future studies.

## Introduction

 Reactive oxygen species (ROS) are molecular oxygen derivatives that arise naturally in aerobic organisms [1]. Under normal physiological conditions, ROS are signaling molecules that participate in regulating cellular signaling pathways, which are critical for proper cellular function and maintenance of homeostasis [2]. However, overproduction of ROS is a factor in the development of a number of diseases, including genetic disorders, cardiovascular diseases, and cancer [2, 3]. In particular, the role of ROS in tumorigenesis has been increasingly investigated [4].

The tumor microenvironment (TME) can promote tumor growth through various mechanisms, such as supplying oxygen and nutrients, secreting tumor-promoting factors and cytokines, and inhibiting immune responses to tumor cells. Conversely, some cells (e.g., natural killer cells, T cells) and regulators (e.g., HSF1, thrombospondin 2, HIF) in the TME possess the ability to eliminate tumor cells [5–7]. As a critical regulator, it is unsurprising that ROS play an intricate role in the tumor-promoting or inhibitory features of the TME [8]. Autophagy also plays a critical role in antigen presentation, which is essential for activation of immune cells, such as T cells. Apoptosis can also modulate immune function by controlling the number and lifespan of immune cells and eliminating autoreactive cells that can cause autoimmune diseases.

Apoptosis is a crucial process for maintaining physical health by eliminating old, unnecessary, and unhealthy cells. Caspases activation, protein and DNA degradation, and membrane alterations that facilitate phagocyte identification are the three main biochemical changes that characterize apoptosis [9]. To better understand the mechanisms and pathways that underlie these changes, two basic apoptotic signaling pathways have been identified: intrinsic and extrinsic. Different intracellular microenvironment perturbations, such as DNA damage, a lack of growth factors, and oxidative stress, activate intrinsic apoptotic pathways. Mitochondrial outer membrane permeabilization (MOMP), a crucial stage in apoptosis, causes the release of several intermembrane space (IMS) proteins, including cytochrome c, to encourage caspase activation [10]. Contrarily, death ligands like FasL, TNF-related apoptosis-inducing ligand (TRAIL), and TNF-α bind to death receptors like Fas, TRAIL-R, and TNF-αR to cause extrinsic apoptosis. This results in the formation of the death-inducing signaling complex (DISC), which then causes the activation of downstream effector caspases [11].

Autophagy, another critical regulator of cell viability, is a catabolic process of phagocytosing cytoplasmic proteins or organelles to degrade cellular contents, ultimately achieving metabolism of the cell itself. Three types of autophagy have been identified: macroautophagy, microautophagy, and partner-mediated autophagy, with macroautophagy being discussed in this review [12]. As mentioned, autophagy is a cellular process that can provide protection and aid in adaptation to stress. However, it is also associated with cell death in various physiological situations. Autophagy-dependent cell death is a form of regulated cell death mediated by the molecular mechanisms of autophagy. In certain conditions, researchers have found that autophagy-dependent cell death occurs in cells that are unable to undergo apoptosis or in cells induced by Tat-Beclin1 [13]. The molecular mechanisms underlying autophagy-dependent cell death in humans are not well understood. To address this, researchers have studied autophagy-dependent cell death in specific tissues of Drosophila, such as the intestine and salivary glands. Evidence suggests that the mechanisms involved in autophagy during cell death differ from those required for cell survival. Studies on Drosophila midgut degradation have revealed that certain components of the autophagic machinery are involved in both autophagy-dependent cell death and general autophagy processes. These components include the initiation complex (Atg1, Atg13, Atg17, and Atg101), PtdIns (3) P-binding proteins (Atg9, Atg2, and Atg18), class III PI3K complex (Vps15 and Vps34), and Atg8a. However, other components, such as Atg7, Atg3, and several other Atg proteins, are not necessary for the cell death process. Ubiquitination, a post-translational modification, plays a role in modulating autophagic flux and is important for tissue-specific autophagy-dependent cell death in Drosophila. Studies on Drosophila salivary glands have identified various signaling pathways that regulate autophagy-dependent cell death, involving histone demethylase Utx, miR-14, Ras-like protein A, Draper (Drpr), and complement-associated macroglobulin (Mcr). Additionally, there are potentially unidentified molecules, including TGF-β, Vps13D, PTP52F, and GBA1, which may be related to autophagy-dependent cell death. During cell death, there is a close interplay between apoptotic and autophagic mechanisms. Apoptotic proteins, including effector caspases, regulate autophagy-dependent cell death, while autophagy-related proteins (Atg) are involved in the clearance of apoptotic cells and have been described as regulators of apoptosis [14].

**Fig. 1:**
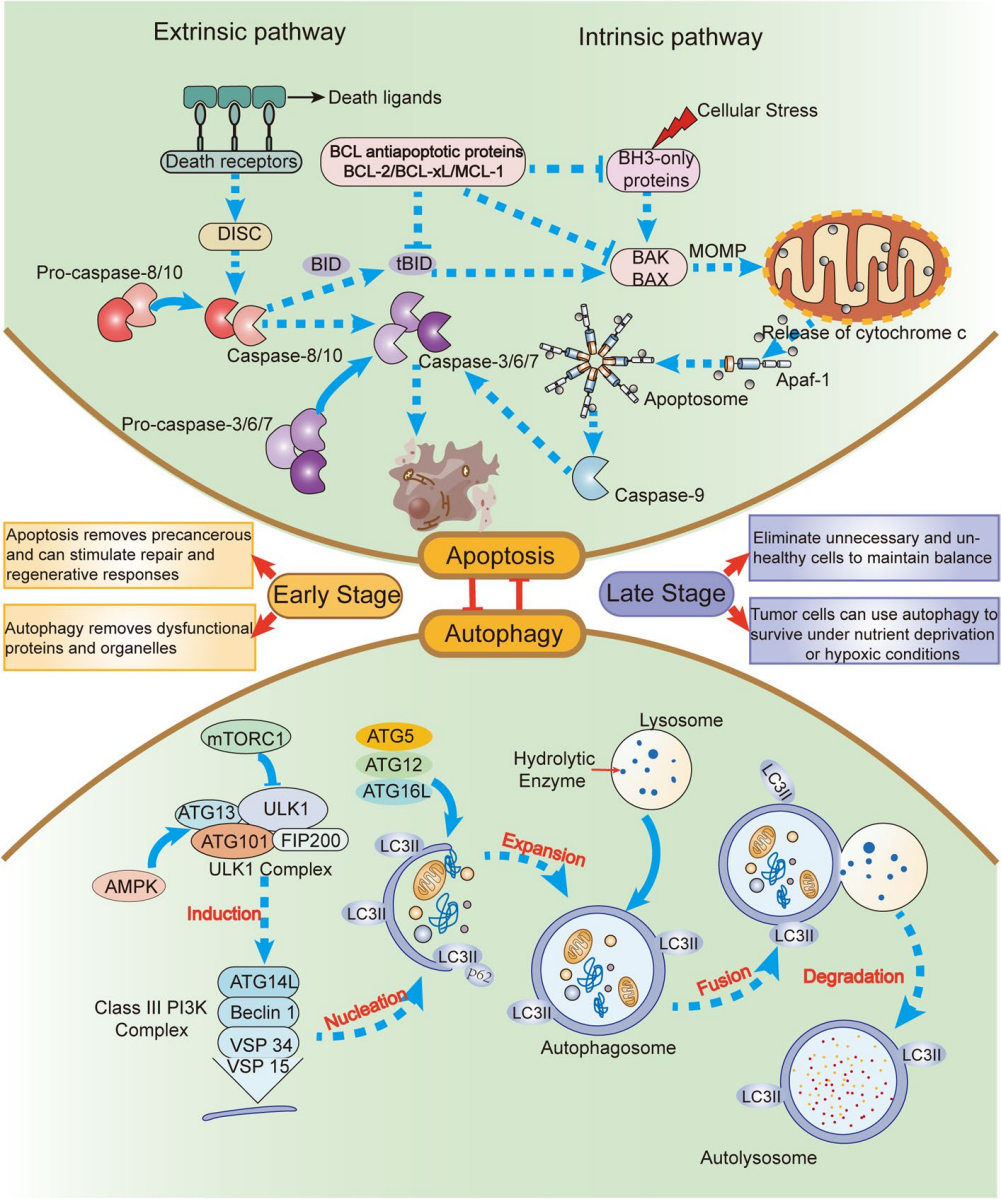
Mechanism and effect of autophagy and apoptosis. Autophagy and apoptosis are two vital mechanisms for maintaining cellular homeostasis under stress conditions. Autophagy is an evolutionarily conserved cellular degradation process that is activated in response to cellular stress signals. The ULK1 complex mediates autophagy initiation, and phagocytic vacuole formation involves the class III phosphoinositide 3-kinase (PI3K) complex, comprising PI3K, ATG14L, Beclin 1, VPS34, and VPS15. The ATG5/ATG12/ATG16 complex and LC3II are subsequently involved, and during autophagosome formation, p62 binds to LC3II while the phagophore expands, encapsulating intracellular material to form autophagosomes. Lysosomes fuse with autophagosomes, providing hydrolytic enzymes for the degradation of phagocytosed material. Two signaling pathways for apoptosis exist: intrinsic and extrinsic. Various intracellular microenvironment perturbations such as DNA damage, growth factor deprivation, and oxidative stress activate the intrinsic apoptotic pathway. Mitochondrial outer membrane permeabilization (MOMP) is a critical step in apoptosis, leading to the release of intermembrane proteins such as cytochrome c. Proteins of the BCL family promote or inhibit this process. Cytochrome c binds to Apaf-1 to form apoptotic bodies, promoting caspase activation. In contrast, extrinsic apoptosis is triggered by the binding of death ligands (e.g., FasL, TNF-α) to death receptors (e.g., Fas, TNF-R), resulting in the assembly of death-inducing signaling complexes (DISC) and the activation of downstream effector caspases (e.g., caspases 3, 6, 7, 8, 10). The two play different roles at different stages

Autophagy has a dual effect on tumor cells. On the one hand, cancer is prevented by autophagy because it eliminates misfolded proteins and malfunctioning organelles, reduces cellular oxidative stress, and eventually stops genetic damage [15]. In general, autophagy inhibition is considered an effective therapeutic strategy to increase tumor cell sensitivity and overcome drug resistance. However, it is crucial to note that if autophagy inhibition or defects in autophagy occur at an early stage, they may not reflect the protective effect of autophagy, but rather promote the occurrence and metastasis of tumors. Studies have demonstrated that the deletion of essential autophagy genes in mice predisposes them to tumor development. In response to stress, autophagy-deficient tumor cells tend to accumulate p62, leading to alterations in NF-κB regulation and gene expression, ultimately promoting tumorigenesis [16]. Additionally, SOCS5, a member of the suppressor of cytokine signaling proteins (SOCS) family, plays significant biological roles in cancer. Overexpression of SOCS5 has been shown to promote tumor development and invasion in vitro through the inactivation of PI3K/Akt/mTOR-mediated autophagy [17]. On the other hand, tumor cells can use autophagy to survive hypoxia or nutritional deprivation in late phases of tumor growth [18]. When stress is too intense or prolonged, autophagy might be blocked and apoptosis can be triggered in some situations [19]. Therefore, autophagy and apoptosis tend to coexist in tumors, and understanding the crosstalk between the two processes can help predict or control cell fate **(**Fig. 1**)**. Currently, there are in-depth studies on the molecular mechanisms linking autophagy and apoptosis, including the interaction between the autophagy-related protein Beclin-1 and the anti-apoptotic protein Bcl-2, as well as the regulation of signaling pathways by protein kinases and transcription factors, which can regulate the switch between autophagy and apoptosis [20–22].

It is clear that the relationship between autophagy and apoptosis in cancer is complex and has significant implications for cancer treatment. One approach is to target both pathways simultaneously, either by promoting autophagic cell death in apoptosis-resistant cancer cells or by inhibiting autophagy in cancer cells that rely on this pathway for survival **(**Fig. 1**)**. Additionally, there is a growing interest in combining cancer therapy with immune-based treatments that can harness the power of the immune system to fight cancer cells. This strategy incorporates immunotherapy, which makes use of medications to activate the immune system to detect and eliminate cancer cells. New research has also focused on the role of oxidative stress in cancer and how it impacts tumor growth and progression. Targeting ROS-induced pathways may be a promising approach to cancer treatment, either alone or in combination with other therapies. Determining how oxidative stress, immunity, autophagy, and apoptosis interact is essential for creating cancer treatments that work.

## Effect of oxidative stress on ROS production in TME

### Reactive oxygen species and oxidative stress

ROS are highly reactive molecules that can cause damage to various biomolecules, including DNA, proteins, and lipids [23]. To maintain redox homeostasis, cells have developed complex antioxidant systems that balance oxidant levels in each cell. However, when this balance is skewed towards oxidation, it is called oxidative stress. In biological systems, redox reactions occur by the transfer of electrons from reduced species to acceptor molecules, ultimately leading to the generation of ROS [24].

**Fig. 2:**
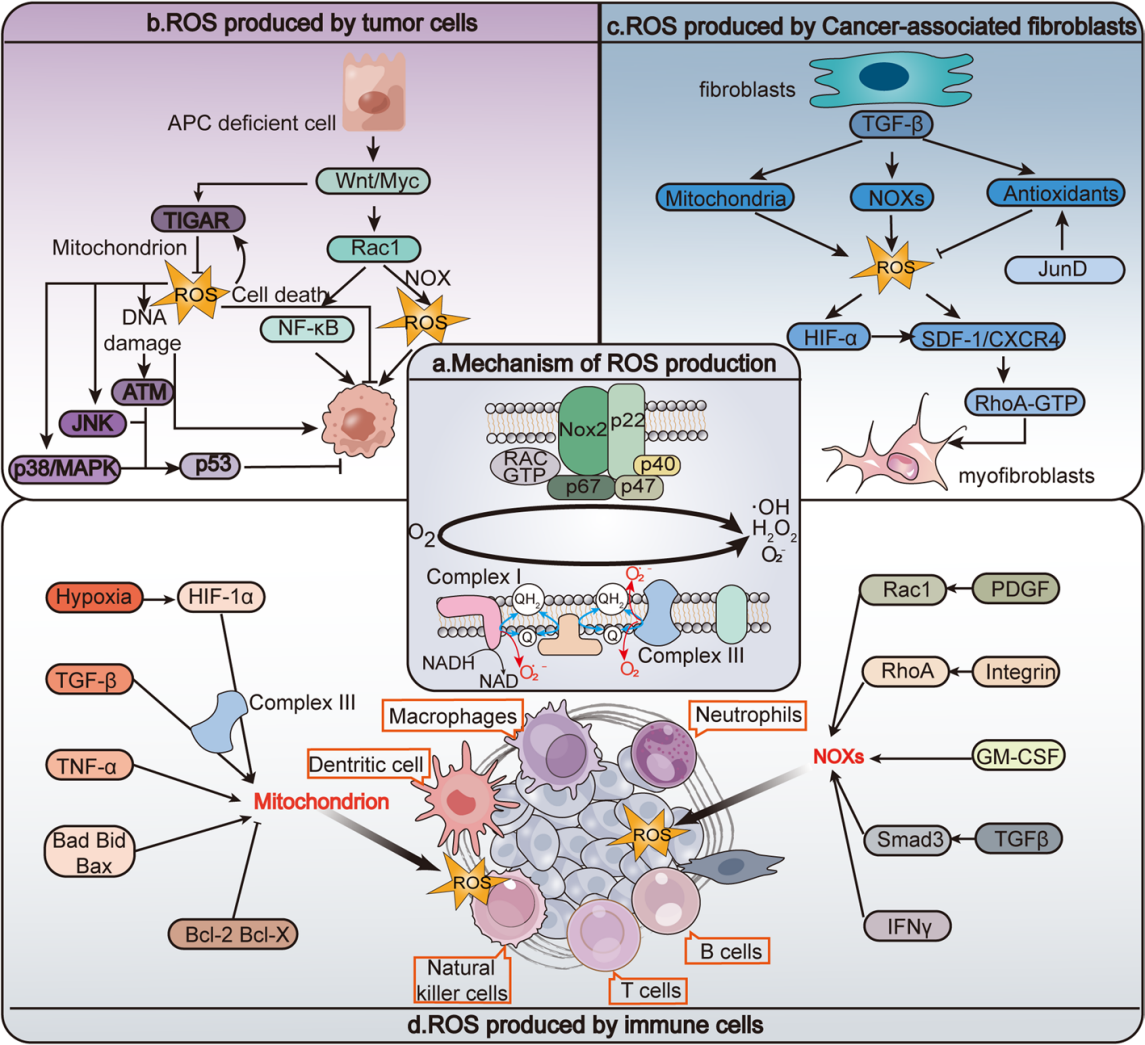
ROS Generation and Signaling Mechanisms in Tumor Microenvironment Dynamics.** a **Mitochondrial ROS are predominantly generated through the mitochondrial electrical transport chain (ETC), primarily complex I (Com I) and Com III. Other mitochondrial enzymes, including NADPH oxidase (NOX), can also directly catalyze ROS generation via enzymatic reactions. NOX proteins require various regulatory subunits, includingp40^phox^,p47^phox^ and p67^phox^, to exert catalytic effects. These subunits have multiple components in the tumor microenvironment, with differing ROS production and action pathways. **b **In APC-deficient colorectal cancer cells, ROS production and NF-κB activation in the NOX pathway, triggered by RAC1, promote WNT-driven intestinal stem cell proliferation and cancer development. The mitochondrial ROS pathway can regulate tumor cell proliferation and growth by activating kinases such as JNK and p38 MAPK, as well as ATM in the DNA damage pathway. **c **Cancer-associated fibroblasts (CAFs) in the tumor microenvironment promote tumor growth and progression and produce ROS through downregulation of mitochondrial electron transport chain, NOX, and antioxidant mechanisms. Oxidative stress can convert fibroblasts into myofibroblasts. ROS mediate TGF-β signaling through various pathways, including redox-dependent accumulation of hypoxia-inducible factor (HIF), stimulating the SDF-1/CXCR4 signaling pathway and activating RhoA-GTPase. Alternatively, ROS can independently stimulate SDF-1, which then causes myofibroblast characteristics in immune cells of TME. **d **ROS production is mainly by one of the two modalities listed above, depending on the cell type, and is regulated by different stimulating factors and signaling pathways. See the figure for a detailed list of these factors and pathways

There are two primary known pathways for ROS production. The first is through the electron transport chain (ETC) of mitochondria, which consists of a transmembrane protein complex (I-IV) embedded within the inner membrane of mitochondria, as well as the freely moving electron transfer carriers ubiquinone and cytochrome c. Complex I (NADH: ubiquinone oxidoreductase) transfers electrons from matrix NADH to ubiquinone, while complex III (coenzyme Q: cytochrome c reductase) transfers electrons from ubiquinone to cytochrome c. Under physiological conditions, some electrons in the ETC may leak directly out of the chain and react with oxygen to produce superoxide [25]. The majority of mitochondrial ROS arises from this process, particularly from complex I and complex III. In addition to respiratory complexes, other mitochondrial enzymes, such as NADPH oxidase (NOX), may directly catalyze the production of O_2_·^−^ or H_2_O_2_ through enzymatic reactions [26]. The types of ROS produced through these pathways include free radicals (such as superoxide anion radical) and non-free radical ROS (such as hydrogen peroxide) [27]. The specific types of ROS produced may vary in different cell tissues **(**Fig. 2a**)**.

In the context of tumors, cancer cells have been shown to have higher levels of ROS than normal cells. Many events that promote tumorigenesis, such as oncogene activation, changes in mitochondrial metabolism, and hypoxia, can increase ROS production. ROS generated by carcinogenic factors may be necessary for tumorigenesis [28]. On one hand, ROS can cause DNA damage and genomic instability that drive carcinogenesis. On one hand, ROS can induce DNA damage and genomic instability, driving the process of carcinogenesis. On the other hand, ROS also oxidatively modify key signaling proteins and proteins involved in crucial biological mechanisms [4, 29, 30]. These modifications play essential roles in the survival, proliferation, metabolism, invasion, and metastasis of cancer cells. The associated signaling pathways mainly include the MAPK pathway, PI3K/AKT/mTOR pathway, NF-κB pathway, and others.

In the MAPK pathway, ROS can activate oncogenic switches, such as HRAS, NRAS, and KRAS, through the oxidation of cysteine residues upstream of the pathway [31]. Furthermore, ROS can activate EGFR and PDGFR signaling in the RAS-guided downstream RAF-MEK-ERK pathway [32, 33]. Another critical signaling pathway in tumorigenesis and metastasis is the PI3K/PTEN pathway. Several critical intermediates in this pathway are highly susceptible to redox dysregulation. ROS can oxidize the cysteine moieties of various phosphatases (PTEN, PTP), leading to their inactivation, which, in turn, facilitates the growth and survival of tumor cells [34]. The transcription factor NF-κB is also affected by ROS, where ROS can influence the activity of IKK upstream of NF-κB. Subsequently, IKK phosphorylates IKB, leading to the release of NF-κB and promoting tumorigenesis [35]. In addition to these signaling pathways, pro-survival autophagy is often activated under stress conditions in tumors, maintaining cellular homeostasis and increasing cell survival. Modification of ROS can alter autophagy in tumor cells through a variety of key autophagy regulatory proteins. For example, Cys105 and Cys113 in p62, a protein linking ubiquitinated substrates to nascent autophagic vesicles, are involved in p62 oligomerization [36]. Furthermore, oxidation of Cys264A to Cys572A in Atg3 or Atg7 mediates the conjugation of LC3 to LC3II, and Cys292 and Cys361 regulate the activity of ATG4B,which can then be decoupled by the above two [37, 38]. Finally, HMGB1, another key regulator of autophagy, undergoes cytosolic translocation stimulated by reactive oxygen species, thereby enhancing autophagic flux. The oxidation of three cysteines (Cys23, Cys45, and Cys106) in HMGB1 was found to be important for their function in regulating autophagy [39].

### Composition of tumor microenvironment

The tumor microenvironment encompasses the internal and external environment of the tumor, including the tissue structure, function, and metabolism as well as the internal environment of the tumor cells themselves. The complexity of the tumor microenvironment is attributed to the presence of various components such as tumor cells, fibroblasts, immune-inflammatory cells, and angiogenesis-related regulators [40]. Cancer cells thrive in low oxygen conditions and undergo metabolic reprogramming to support their energy and nutrient demands for survival and proliferation. Cancer cells are distinguished by this metabolic reprogramming, which causes an excessive generation of ROS and other metabolites [41, 42].

Changes in the tumor microenvironment, such as remodeling and alterations in cell signaling, can promote tumor growth and transform normal cells into malignant cells [43, 44]. ROS, considered important signaling molecules and metabolites, are produced in excessive amounts due to an imbalance between ROS production and antioxidant system (AOS) activity, which is observed in many types of cancer. Changes in protein quality control systems, signal transduction pathways, and gene expression levels that affect cancer cell proliferation, migration, invasion, and apoptosis result from this [45–48]. The next sections will discuss the mechanisms and effects of ROS production by various components in the tumor microenvironment.

### ROS producers in TME: from induction to accumulation

#### ROS produced by tumor cells

Increased levels of ROS in cancer cells can be attributed to reduced antioxidant enzymes and increased production of intracellular oxidants such as mitochondria, NOX, and cyclooxygenase (COX) [49]. Mitochondria and NADPH oxidase are the major sources of endogenous ROS in cancer, and recent studies have revealed a crosstalk between these two producers, referred to as “ROS-induced ROS release”. For instance, induced mitochondrial superoxide, hydrogen peroxide, and peroxynitrite can trigger activation of NADPH oxidase in isolated leukocytes [50]. Oncogenes such as MYC have also been reported to enhance ROS generation, leading to DNA damage and impaired p53 function [51]. Furthermore, the Warburg effect promotes alterations in mitochondrial redox potential, which eventually alter ROS production and increase oncogenic activity [52].

Excessive ROS production in cancer cells induces several biological effects. Firstly, as control mechanisms for cell proliferation, ROS directly interact with certain receptors, and the redox status of signaling molecules such protein kinases and transcription factors is altered, including the growth factor receptors mentioned above, MAPK, PI3K, NF-κB, etc. Secondly, high levels of ROS lead to genomic instability through oxidative damage to DNA, which is a major driving force in tumorigenesis [53]. Finally, ROS overproduction leads to cell damage and cell death [54]. It has been shown that ROS is required for cell proliferation in colorectal cancer, where RAC1-triggered ROS production and NF-κB activation promote WNT-driven intestinal stem cell proliferation and cancer development [55]. DNA damage is also an important factor in inducing cancer. DNA-damaging agents, such as H_2_O_2_, can activate stress-activated kinases (SAPKs), including JNK, p38, and MAPK, as well as important kinases (including ATM and ATR) involved in the DNA damage response [56]. Additionally, ROS are a mediator of cell death. For example, ROS production drives WNT-mediated proliferation of intestinal cells, and it has been demonstrated that TIGAR and RAC1-derived ROS can have diametrically opposite effects on Wnt-driven intestinal proliferation in mice, with ROS exhibiting both proliferative and antiproliferative effects [57]. The final outcome is dependent on the type of ROS involved as well as the level of ROS exposure **(**Fig. 2b**)**.

#### ROS produced by Immune cells

The tumor microenvironment is home to a variety of immune cells, including T cells, B cells, NK cells, and bone marrow-derived cells, such as neutrophils, macrophages, dendritic cells, and myeloid-derived suppressor cells. These immune cells play a critical role in the development, progression, treatment, and prognosis of cancer [58]. In addition, there may be differences in the mechanisms, pathways, and stimulating factors involved in ROS production by immune cells under normal immune conditions versus pathological conditions. Below we describe in detail how tumor-associated immune cells produce ROS.

In normal immune conditions, ROS are essential for proper immune cell function. The NADPH oxidase complex consists of multiple components, including transmembrane xanthocytochrome b_558_. These components remain physically dissociated in the inactive state in the absence of infection. However, when regulatory subunits are upon activation, they translocate to the membrane and bind energetically to xanthocytochrome b_558_ [59]. Various internal and external stimuli, such as chemokines, cytokines, growth factors, phagocytosis, cell adhesion, and activation of cellular receptors, induce NOXs at the molecular level [60, 61]. In tumor tissue, signaling molecules such as platelet-derived growth factor (PDGF), integrin, GM-CSF, IFN-γ, and TGF-β, which are involved in tumor growth, spread, and metastasis, can promote or restrain NOX-mediated ROS production in tumor-associated immune cells [58].

With the use of fluorescence resonance energy transfer (FRET), a biosensor that can selectively track ROS production at focal adhesion (FA) sites in living cells has been created. The findings demonstrated that Rac1 activation by PDGF caused ROS generation at FA locations [62]. Through RhoA activity, integrins, in particular integrin αvβ3, can reduce this local ROS production. Responses to tumor pathogenesis depend heavily on intercellular communication between tumor cells, bone marrow cells, and T lymphocytes. GM-CSF upregulates NOX2 expression and releases ROS into granulocytes, which suppresses T cells and causes tumor development [63]. Additionally, NOX has been reported to be TGFβ-Smads dependent. For example, NOX4 is involved in TGFβ-Smad3-regulated epithelial-mesenchymal transition in breast cancer and also provides a source of ROS for Epithelial-to-mesenchymal transition (EMT) phenotypic switching in pancreatic cancer [64, 65].

The production and release of ROS into the cytoplasm of mitochondria can be impacted by a number of things. These factors include hypoxia, TNF-α, TGF-β, pro-apoptotic proteins, and anti-apoptotic proteins of the Bcl-2 family [58]. TGF-β can influence ROS generation by affecting NOX and mitochondrial ROS regulation. TGF-β activates complex III of the electron transport chain to increase the generation of ROS in the mitochondria, and TGF-β-induced NOX4 transcription requires ROS production from mitochondria, which creates a feed-forward loop that amplifies intracellular ROS signaling following TGF-β activation [66]. ROS are crucial signaling pathway moderators and can regulate TNF-α triggered apoptotic signaling and NF-κB transcription, which can lead to simultaneous activation of apoptotic and survival pathways mediated by NF-κB transcription upon TNF-α activation [67]. In this study, we further confirmed the direct association of TNF-α-induced ROS production with ROS modulators 1 (Romo1) and B-cell lymphoma-extra large (Bcl-XL) [68]. Hypoxia is often present in the tumor microenvironment, which increases the stability of hypoxia-inducible factors (HIFs). Under hypoxia, mitochondrial ROS release stabilizes HIF-1α by a mechanism dependent on respiratory chain complex III **(**Fig. 2d**)** [69].

#### ROS produced by Cancer-associated fibroblasts

Cancer-associated fibroblasts (CAFs) are stromal cells present in the tumor microenvironment that can promote tumor growth and progression. CAFs are a heterogeneous group of cells that vary in their origin, phenotype, function, and abundance across different cancer types [70]. These cells can originate from various sources, including endothelium, bone marrow cells, vascular pericytes, adipocytes, and local resident fibroblasts [71–73]. Oxidative stress can lead to the conversion of fibroblasts into myofibroblasts, which play a significant part in shaping the heterogeneous tumor microenvironment. ROS are produced in a similar manner as described earlier, and one of their main functions is to transform fibroblasts into myofibroblasts through various signaling pathways. For instance, two autocrine signaling loops that act in an autologous stimulation and cross-communication manner are transforming growth factor β1 (TGF-β1) and stromal cell-derived factor 1 (SDF-1) [73]. The activation of TGF-β1 requires ROS produced by mitochondria, which mediate TGF-β signaling through different pathways, including the SMAD pathway, MAPK pathway, and Rho-GTPase pathway [74]. SDF-1 can also induce fibroblast-to-myofibroblast transformation in a ROS-dependent manner [75]. Other factors, such as redox-dependent accumulation of HIF, can stimulate the CXCL12/CXCR4 signaling pathway and activate RhoA-GTPase, eliciting myofibroblast characteristics **(**Fig. 2c**)** [76].

## Regulation of crosstalk between autophagy and apoptosis in oxidative stress

Various signal transduction pathways and molecules triggered by cellular stress can regulate both autophagy and apoptosis. ROS are associated with these pathways and mediate the crosstalk between autophagy and apoptosis. In some cases, these pathways are co-excited or inhibited, while in others, they show mutual inhibition.

Intracellular ROS production can have a dual effect on apoptosis. By activating pro-apoptotic proteins and inhibiting anti-apoptotic proteins, it may, on the one hand, enhance the apoptotic process. However, it might also activate associated products to prevent apoptosis. Common death-inhibiting proteins, such as FLIP and XIAP, play a crucial role in cell fate determination by regulating apoptosis-promoting proteins, including ASK1, AKT, Bax, Bak, caspase-9, and Apaf-1. Increased ROS can also activate anti-apoptotic pathways in the body, such as the transcription factor NF-kB. These apoptotic effectors are all redox-sensitive, and their regulation is selective; the same protein can either be activated or inhibited by redox alterations.

The redox-sensitive signals affecting cell death/survival are integrated, and cell survival or apoptosis depends on their balance. Mild ROS stress can lead to anti-apoptotic signals and cell survival, while severe ROS stress can promote apoptosis. The functions of the substances involved in apoptosis and their direct redox regulation by ROS are summarized in Table 1.

**Table 1:** Molecular and functional pathways involved in oxidative regulation of apoptosis

Components involved in apoptosis	Basal function	Effects after oxidative regulation	Ref
**Transcriptional level**			
NF-κB	NF-κB as a transcription factor activates transcription of target genes, such as elevated expression of anti-apoptotic Bcl2 family (Bcl-XL), FLIP, caspase inhibitors (XIAP)	ROS↑ activation of NF-κB↑ anti-apoptotic protein expression ↑	[77]
Nrf2	Nrf2 regulates the transcription of genes, the protein of these genes have antioxidant and glutathione synthase functions and can defend against oxidative stress.	ROS↑ dissociation of the Nrf2 – KEAP1 complex↑ Activation of Nrf2↑ ROS↑ phosphorylation of PKC↑ Activation of Nrf2↑	[78]
FOXO	It stimulates transcription of genes for antioxidant proteins located in different subcellular compartments, (mitochondria, peroxisomes and plasma)	ROS can modulate FOXO activity at multiple levels post-translational modifications of FOXO (e.g., phosphorylation and acetylation), interactions with co-regulators, changes in FOXO subcellular localization	[79]
HIF	In most cases, HIF promotes cell survival through transcriptional regulation of angiogenic factors and glycolytic enzymes HIF may induce apoptosis by increasing the expression of pro-apoptotic factors such as NOXA, BNIP3, and Nix	ROS↑ PI3K/Akt and p38 MAPK↑ S-nitrosylation of HIF↑ HIF↑	[80]
P53	P53 can directly activate pro-apoptotic Bcl-2 family members (Bax and Bak) with the Bcl-2 family, thereby inducing mitochondrial outer membrane permeability (MOMP) and apoptosis	ROS can regulate p53 function not only by direct oxidative modification, but also indirectly by ATM or p38 MARK	[81]
**Signalling transduction level**			
PI3K/AKT	Act as anti-apoptotic pathway to a variety of stimuli such as radiation, hypoxia by phosphorylation and inactivation of pro-apoptotic proteins	ROS modifies PTPases and PTEN PI3K↑ ROS modifies PI3K and Akt PI3K and Akt↓ survival signaling↓	[82]
MAPK(ASK1)	Under non-stress conditions, ASK1 activity is blocked when combined with Trx-1	ROS↑ Trx-1 oxidation and release of ASK1↑ Activation of JNK and p38-MAPK↑	[83]
AMPK	AMPK acts as an energy sensing factor that links metabolism and maintains redox balance	AMP/ATP ratio↑ AMPK phosphorylation↑ ROS↑ AMPK↑(via S-glutamylation of cysteine on the α and β subunits of AMPK)	[48]
**Executive level**			
FLIP	Bind to FADD ,caspase-8/10 and DR5 and form an AIC AIC prevents the formation of the DISC and activation of the caspase cascade	ROS↑ c-FLIP↓ cFLIP levels correlate with the proportion of different intracellular species of ROS, such as O_2_·^-^ and H_2_O_2_	[84, 85]
XIAP	Bind and inhibit caspases 3, 7 and 9	H_2_O_2_↑ PI3K/Akt phosphorylation↓ link between XIAP and Akt↓ XIAP↓	[86, 87]
Cytochrome c	Involve in oxidative phosphorylation and ATP production as a component of mitochondrial ETC	1)ROS↑ intracellular Ca^2+^↑ nonspecific pores (MPT pores) ↑ OMMP↑ 2)ROS↑ the activation of Bcl-2 family (Bax and Bak) by regulating ASK1/JNK pathway OMMP↑	[88–90]
caspase9 and Apaf-1	Apaf-1 and caspase 9 together with cytochrome c form a complex (apoptosome)	ROS (H_2_O_2_)↑ caspase-9 activation↑	[91, 92]
Alox5	Under caspase-9-deficient condition, Erk1-Alox5 is a potentially important signaling pathway to execute both apoptotic and nonapoptotic forms of cell death	ROS↑ Alox5-mediated lipid peroxidation↑ nuclear entry of cell death-inducing molecules EndoG and TIA-1↑	[93]

*Abbreviations*: *NF-κB *Nuclear factor kappa-light-chain-enhancer of activated B cells, *Bcl2 *B-cell lymphoma 2, *FLIP *FLICE-like inhibitor protein, *XIAP *X-linked inhibitor of apoptosis protein, *Nrf2 *Nuclear factor erythroid 2-related factor 2, *KEAP1 *Kelch-like ECH-associated protein 1, *PKC *Protein kinase C, *PI3K *Phosphoinositide 3-kinases, *AKT *Protein kinase B, *PTEN *Phosphatase and tensin homolog, *ASK1 *Apoptosis signal regulating kinase-1, *MAPK *Mitogen-activated protein kinase, *TRX-1 *Thioredoxin-1, *JNK *c-Jun N-terminal kinase, *AMPK *5’ AMP-activated protein kinase, *FADD *Fas-associating protein with a novel death domain, *DR5 *TRAIL receptor 5, *AIC *Apoptosis inhibitory complex, *DISC *Death-inducing signaling complex, *OMMP *Outer mitochondrial membrane pores, *Apaf1 *Apoptotic protease activating factor 1, *EndoG *Endonuclease G, *Alox5 *Arachidonate 5-lipoxygenase, *Erk1 *Extracellular signal-regulated kinases

The cellular process of autophagy, which is strictly controlled, is essential to the stress response. A growing body of research indicates that ROS contribute to the stimulation of autophagy during the initiation and progression of cancer. All phases of autophagy, including initiation, extension, fusion, and degradation, are subject to oxidative regulation, and changes in important metabolic pathways that trigger autophagy are strongly redox-dependent.

mTOR is a critical regulatory molecule that activates autophagy during the initiation phase. Autophagy is inhibited when mTOR is activated, but autophagy is promoted when mTOR is negatively regulated. Both ULK1 and ULK2 act as mammalian functional homologs of yeast Atg1 and play a central role in starvation-induced autophagy. The ULK1 complex translates signals from upstream autophagic pathways into downstream mTOR and AMPK signals by integrating data from upstream sensors. ROS have stimulatory effects on upstream sensors. PI3K Class III complexes, containing hVps34, Beclin-1, p150, and Atg14-like proteins, are required for the induction of autophagy. ROS regulates Beclin 1 protein dissociation. During the elongation phase, autophagosome formation is controlled by regulation of Atg12-Atg5 as well as LC3-II (Atg8-II) complexes. However, the fusion and degradation process remains unclear and requires further investigation. These mechanisms and influencing factors are summarized in detail in Table 2.

**Table 2:** Molecular and functional pathways involved in oxidative regulation of autophagy

Components involved in autophagy	Basal function	Effects after oxidative regulation	Ref.
**Initiation**			
AMPK	AMPK, a key factor regulating autophagy, is a major sensor of intracellular energy stress and is able to sense and respond to energy changes and is essential for energy homeostasis	ROS↑ AMPK↑ activating Ulk1 through phosphorylation of Ser 317 and Ser 777 ↑ autophagy↑	[94]
mTOR	mTOR is a negative regulator of autophagy. It impedes ULK1 complex formation by phosphorylating ULK1 and Atg13 proteins in components of the ULK1	1)ROS↑ AMPK↑ Phosphorylation of TSC2 and RAPTOR↑ mTORC1 inhibition↑ autophagy↑ 2)ROS↑ oxidization of PTEN↑ PI3K/AKT↑TSC1 / 2↓ mTORC1 inhibition↑ autophagy↑	[95, 96]
Beclin1	BCL-2 or PI3k class III play critical roles in the regulation of autophagy and cell death through binding interactions with Beclin1	1)ROS↑ JNK↑ Bcl2 phosphorylation↑ BECN1 release from the inhibitory BCL2-BECN1 complex autophagy↑ 2) ROS↑ stability of HIF↑ BNip3↑ BNip3 regulate autophagy by competing with Beclin-1 for binding to Bcl2, thereby releasing Beclin-1 to induce autophagy	[97, 98]
**Expansion**			
Atg5-Atg12-Atg16 complex	The Atg5-Atg12-Atg16 complex acts as an ubiquitin-like conjugation system and contributes to elongation and autophagosome maturation of isolated membranes	ROS↑ Atg5 Atg12↑ autophagy↑	[99, 100]
LC3/LC3-I/LC3-II	LC3-I is produced after LC3 is cleaved by Atg4 LC3-I binds to phosphatidylethanolamine (PE) to form a lipidated form of LC3-II through an ubiquitin-like reaction that requires the participation of Atg7 and Atg3	Mild level of ROS induce ATG3 and ATG7 oxidation, contributing to inhibition of LC3 lipidation. High level of ROS lead to ATG4 oxidation and inhibit its LC3 decoupling function, thereby allowing phagophore expansion. ATG3 and ATG7 may be more sensitive than ATG4.	[101–103]
SQSTM1	SQSTM1 achieves autophagic cargo recognition by interacting with LC3, involved in autophagic cargo assembly and autophagosome-lysosome fusion. The antioxidant effect of SQSTM1 is mainly achieved through its activation of NRF2 and NFKB1	Mild ROS increases, SQSTM1 first uses autophagy to recognize LC3 Severe ROS increases, autophagy antioxidant defenses all fail to limit ROS, and SQSTM1 activates NFE2L2 or NFKB1 antioxidant gene transcription	[104]
**Fusion**			
SNARE System (STX17,SNAP29,VAMP8/7)	It is involved in autophagosome-lysosome fusion as well as other autophagic processes, including autophagosome formation and mitophagy	?ROS↑ SNAR depletion leads to accumulation of autophagosomes without degradation	[105, 106]
TRPML1	It mediates lysosomal enzyme calcium release and activation triggers calcineurin-dependent TFEB nuclear translocation, autophagy induction and lysosomal biogenesis	TRPML1 is specifically required for ROS-induced autophagy, with oxidants specifically activating lysosomal TRPML1 channels and calcium release	[107]
**Degradation**			
Lysosomal protease	Autophagosomes combine with lysosomes to form autolysosomes, and the contents in autolysosomes decrease in the action of enzymes.	Lysosomal protease cathepsin has different sensitivity to ROS, with increased ROS and decreased hydrolytic activity of the enzyme	[107]

*Abbreviations*: *AMPK *5’ AMP-activated protein kinase, *Ulk *Unc-51 like autophagy activating kinase, *Atg *autophagy related gene, *TSC1/2 *Tuberous sclerosis 1/2, *BNip3 *BCL2/adenovirus E1B 19 kDa protein-interacting protein 3, *LC3 *Microtubule-associated proteins 1 A/1B light chain 3B, *SQSTM1 *Sequestosome-1, *SNARE *Snap receptor, *STX17 *Syntaxin 17, *SNAP29 *Synaptosomal-associated protein 29, *VAMP8/7 *Vesicle-associated membrane protein 8, *TRPML1 *Transient receptor potential cation channel, mucolipin subfamily, member 1, *TFEB *Transcription factor EB

### ROS regulates the production and modification of transcription factors

Transcription factors such as NRF2, NF-κB, and p53 play important roles in regulating the shift of cell fate towards autophagy or apoptosis.

NRF2 is a crucial transcription factor that helps maintain redox homeostasis. It is a potential target for cancer treatment, with resveratrol and rutine being the focus of current studies [108]. In response to low levels of oxidative stress, NRF2 is activated by ROS and participates in regulating both autophagy and apoptosis. Normally, NRF2 is held in complex with KEAP1, but oxidative stress can modify cysteine thiols in both proteins, leading to structural changes that release NRF2. When NRF2 is unbound, it moves to the nucleus and interacts with antioxidant response elements (ARE) and initiates transcription of antioxidant genes that help reduce apoptosis [109]. Additionally, when NRF2 is in the nucleus, it also activates the transcription of autophagy-related genes through the binding of small Mafs proteins to ARE, which promotes the process of autophagy [110, 111]. However, paradoxically, research has shown that an increase in NRF2 levels can negatively regulate autophagy, and the underlying mechanism remains poorly understood and requires further investigation [112].

NF-κB, one of the first transcription factors that free radicals have been demonstrated to control, can also be activated directly by different species of ROS. Inhibition of NF-κB accumulates reactive oxygen species, which inactivate MKPs to promote JNK activation and apoptosis. Different species of ROS can also directly activate NF-κB, which in turn promotes autophagy by up-regulating the expression of LC3 and the important autophagy genes ATG5 and BECN1. The autophagy pathway is involved in the inhibition of NF-κB-dependent apoptotic responses [113].

P53 is a crucial tumor suppressor and apoptosis-inducing factor that stimulates antioxidant pathways and autophagy. Accumulation of ROS activates transcription factors that inhibit mTOR, such as AMPK/TSC1/2, which in turn regulates autophagy. In order to cause mitochondrial outer membrane permeability (MOMP) and apoptosis, P53 can interact with the pro-apoptotic Bcl-2 family members (Bax and Bak) as well as the anti-apoptotic Bcl-2 family (Bcl-2, Bcl-XL). Furthermore, p53 participates in the transcriptional activation of genes related to autophagy, including DRAM (injury-regulated regulator of autophagy) [114, 115].

Furthermore, there are some important non-transcriptional associated factors, such as HMGB1. High mobility group box 1 (HMGB1) is a protein that controls autophagy in a ROS-dependent way and is thought to be a damage-associated molecular pattern protein. ROS encourage HMGB1 to move from the nucleus to the cytoplasm, where it binds to Beclin1 directly and triggers autophagy. Inhibition of autophagy to reduce HMGB1 release can enhance apoptosis. Activated oxidized HMGB1 caspases subsequently induce mitochondrial apoptotic pathways [116].

### ROS-regulated protein kinase signal transduction pathways

Common components of the ROS-regulated protein kinase transduction pathway include AMPK, AKT, and MAPK. ROS regulate autophagy and apoptosis by controlling signaling pathways and regulating the activity of kinases. Elevated ROS levels can modulate the regulation of kinase activity and its activation of AKT, AMPK, and ASK in autophagy and apoptosis crosstalk. Under hypoxic conditions, ROS can directly trigger AMPK or activate it via ROS-dependent calcium channel activation. Inhibition of downstream mTOR kinase activity promotes autophagy, which removes damaged protein aggregates and organelles, thereby reducing oxidative stress-mediated apoptotic responses [94]. ROS can also inactivate PTEN, leading to AKT activation and inhibition of apoptosis. Akt kinase signaling cascades activate mTOR and inactivate BECN1 to inhibit autophagy [113]. In the MAPK cascade, DJ-1 acts as a redox sensor and senses ROS levels. Under mild oxidative stress, DJ-1 inhibits ASK1 activity and activates autophagy to maintain cell viability. However, under severe oxidative stress, hyperoxidized DJ-1 dissociates and activates ASK1, which then activates p38 and JNK, promoting apoptosis. JNK inactivates the anti-apoptotic Bcl-2 family, further promoting apoptosis [117].

## Control of immunity by ROS

### Regulation of innate immunity

Innate immune responses are the first line of defense against pathogens or danger signals, and are characterized by their rapid response. Activation of innate immunity promotes phagocytosis, secretion of active factors, and can indirectly activate antigen-presenting cells (APCs), which in turn promote adaptive immune responses [118]. During an immune response, immune cells undergo metabolic reprogramming, which can increase cellular ROS production and contribute to functional changes in immune cells. NOX and mitochondria are the two main sources of increased ROS production in innate immune cells.

**Fig. 3:**
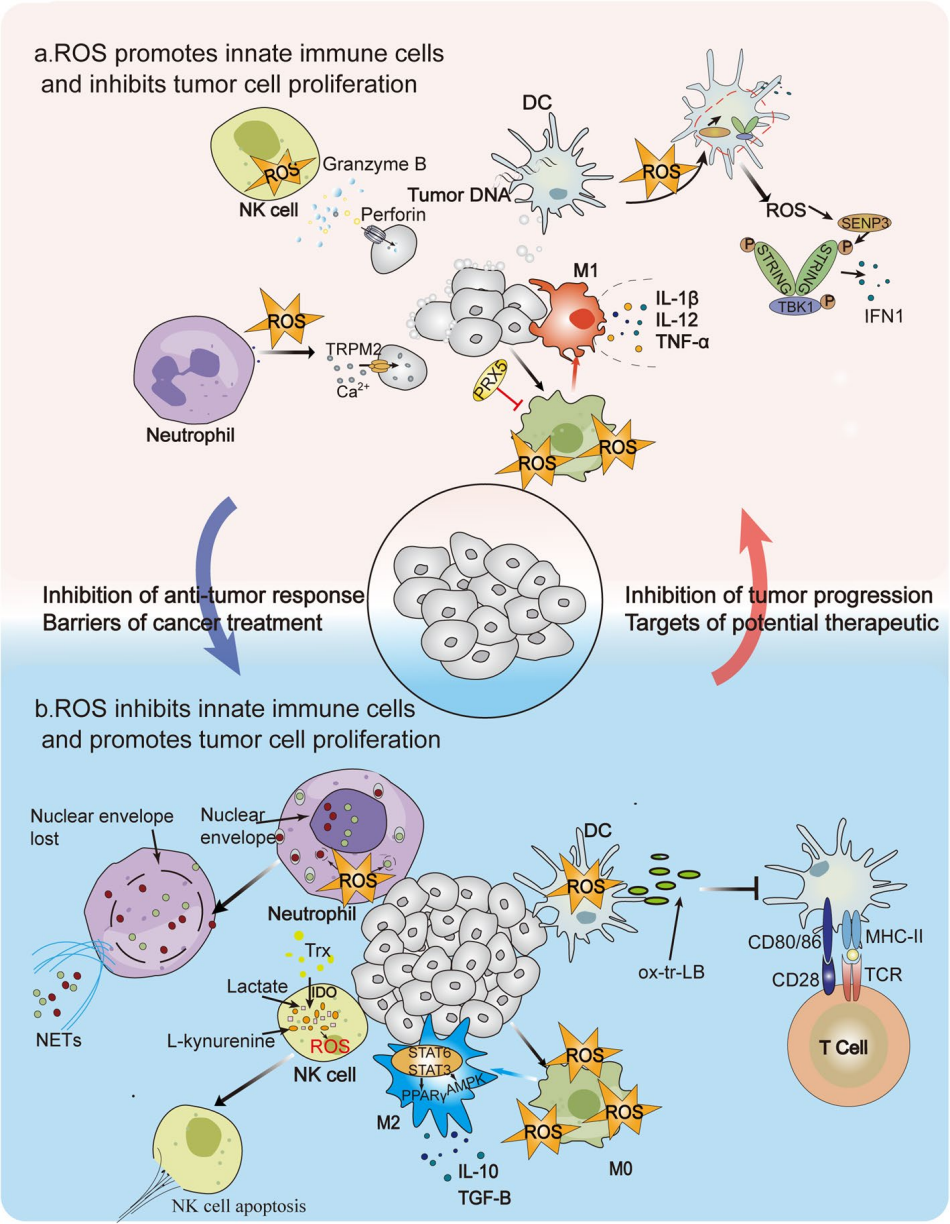
Role of ROS in innate immunity. ROS have a dual role in innate immunity and can function as either a potential therapeutic target against cancer by inhibiting tumor cell proliferation and promoting innate immune cells or as an obstacle to treatment by promoting tumor cell proliferation and inhibiting innate immune cells. **a** Promotion. ROS contribute to macrophages becoming M1 phenotype and mediating the phagocytic activity of M1 macrophages by releasing factors such as IL-1, IL-12, and TNF-α. Neutrophils play a crucial role in killing tumor cells and preventing tumor spread and metastasis. ROS generation induces excessive calcium influx in tumor cells through TRPM2 channels, inhibiting tumor cell growth and metastasis and driving the cytotoxic effect of neutrophils. ROS production is an early event after NK cells recognize cancer cells and induce the production of cytotoxic essential substances such as perforin and granzyme. ROS generation in DC cells regulates their phagocytic activity and maintains alkalinization. When tumor cells enter DCs, the activation of STING by tumor cell DNA promotes the production of IFN1 and other defense cytokines. **b** Suppression. ROS promote macrophage transformation to M2 type and secrete inhibitory cytokines such as IL-10 and TGF-β, which have pro-angiogenic and immunosuppressive functions and promote tumorigenesis. ROS also promote the generation of neutrophil extracellular traps (NETs), which can have both tumor-promoting and metastasis-promoting effects. High ROS levels in the TME may also be detrimental to NK cell survival, and L-kynurenine as well as lactate produced by IDO can lead to NK cell apoptosis through ROS pathways in NK cells. Additionally, excess ROS can oxidize lipids and form lipid bodies (LB) of electrophilic oxidized truncated (ox-tr) lipids, inhibiting antigen presentation by DCs to T cells and weakening immune responses

ROS generated by NOX2 complexes can directly oxidize proteins, DNA, and carbohydrates, thereby killing pathogens. Recognition of pathogen-associated pattern molecules (PAMPs) and damage-associated pattern molecules (DAMPs) by innate immune cells leads to increased ROS generation, inflammasome activation, and pro-inflammatory cytokine production [119]. Elevated ROS also mediate the primary antimicrobial response of phagocytes, known as the respiratory burst, which is required for clearance by phagocytes such as monocytes, macrophages, and neutrophils [120]. The regulation of ROS on innate immune responses in the context of cancer is elaborated on below, starting from specific innate immune cells (Fig. 3).

Neutrophils play a crucial role in killing tumor cells and preventing tumor spread and metastasis. Studies have shown that ROS production, particularly H_2_O_2_, is a major factor driving the direct cytotoxicity of neutrophils towards tumor cells. H_2_O_2_ inhibits tumor cell growth and metastasis by inducing excess calcium influx in tumor cells through TRPM2 channels [121]. In addition to the direct killing effect of neutrophils, the role of neutrophil extracellular traps (NETs) in tumor progression remains ambiguous. While some studies suggest that NETs have tumor-promoting and metastasis-promoting effects, conflicting views exist regarding their ability to induce apoptosis and inhibit tumor growth [122, 123]. NETs are composed of chromatin-bound DNA decorated with proteins such as myeloperoxidase (MPO) and neutrophil elastase (NE) [124]. The formation of NETs is induced within primary tumors and requires ROS-mediated citrullination of histones.

NK cells play an essential role in innate immunity and are capable of killing tumor cells through various mechanisms. ROS generation is an early event following NK cell encounter with cancer cells, and NK cell-mediated lysis involves hydroxyl radical-dependent steps [125]. However, the high level of ROS in the tumor microenvironment can be detrimental to NK cell survival and function during the subsequent course of tumor development. Specifically, L-kynurenine and lactate produced by IDO can lead to NK cell apoptosis through ROS pathways [126, 127]. To counteract this, activation of antioxidant pathways, including thioredoxin, can increase NK resistance to oxidative stress [128].

Macrophages are a heterogeneous population of cells that can be divided into M1 and M2. By engulfing cancer cells, secreting huge amounts of pro-inflammatory cytokines like IL-1, IL-12, and TNF-α, and luring T cells, M1 macrophages perform a pro-inflammatory function and take part in immune surveillance. M2 macrophages, in contrast, release inhibitory cytokines like IL-10 and TGF-β, which suppress immune responses and aid in tissue remodeling and inflammation eradication [129]. Tumor-associated macrophages (TAMs) have both pro-angiogenic and immunosuppressive functions, which promote tumorigenesis, as well as tumor suppressive effects, similar to the characteristics of M1 macrophages. Thus, TAMs are not a single macrophage population but may have both M1 and M2 phenotypes [130]. In the polarization of macrophages, ROS are crucial [131]. Numerous studies have suggested that ROS are necessary for TAMs to invade tumors and develop an M2 phenotype that promotes tumor growth. For instance, H_2_O_2_ inhibits the production of pro-inflammatory cytokines like TNF-α and promotes, via STAT6, the transcription of pro-fibrotic proteins, decreasing the M1 phenotype and activating the M2 phenotype [132]. Additionally, ROS are involved in regulating the phagocytic activity and inflammatory response responsible for the M1 phenotype. In order to mediate phagocytic activity in M1 macrophages, stimulation of mitochondrial ROS generation through the inner mitochondrial membrane’s electron transport chain is important [133].

In recent studies, ROS regulation of macrophages has been recognized as a promising strategy to combat clinically refractory cancer. Repolarization of M2 to immune-promoting M1 type by regulating oxidative stress is an increasingly considered approach [134]. For instance, the ROS inhibitor BHA has been reported to significantly inhibit tumorigenesis by abrogating ROS and blocking macrophage differentiation to M2 in several carcinogen-induced mouse tumor models [135].

Dendritic cells (DCs) are highly capable of acquiring, processing, and presenting antigens, and have been used in clinical immunotherapy to eliminate tumor cells by harnessing the patient’s own immune system. The ability of DCs to present antigens and activate T cells is influenced by their exposure to ROS in the environment. To initiate an adaptive cytotoxic immune response, DCs present proteolytic peptides derived from phagocytic antigens to CD8^+^ T lymphocytes through a process called antigen “cross-presentation,“ which relies on the activity of NOX2 and the generation of ROS to maintain an alkaline environment [136]. When tumor cells enter DCs, some tumor cell DNA may enter the cytoplasm and activate STING, an endoplasmic reticulum (ER)-related protein, promoting the production of type I IFN and other cytokines involved in host defense. This is critical for DC-mediated immunogenic tumor T cell-driven immune responses. Stimulation of STING in a ROS-dependent manner favors intrinsic antitumor immunity and cancer immunotherapy [137]. However, ROS has a dual effect. Senescent DCs may interfere with cross-presentation by producing ROS, which decreases Δψm and ATP. Excess oxidation of intracellular lipids produces reactive by-products that can lead to ER stress responses and the formation of lipid bodies (LB) containing electrophilic oxidized truncated (ox-tr) lipids, thereby inhibiting antigen presentation by DCs to intratumoral T cells [138, 139].

### Regulation of adaptive immunity

**Fig. 4:**
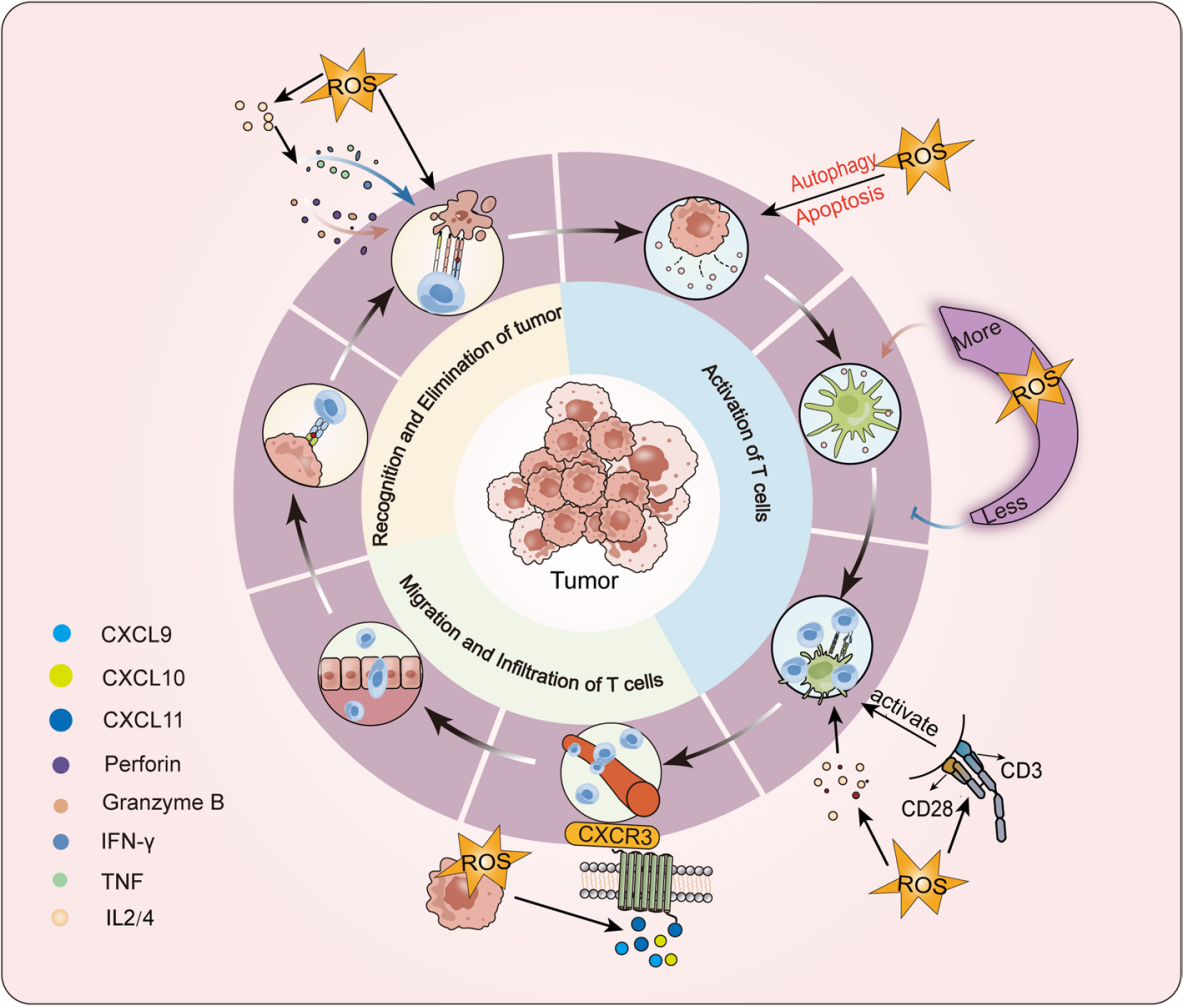
Role of ROS in adaptive immunity. Adaptive immunity in tumors involves the capture, processing, and presentation of antigens by DCs, leading to the activation of effector T cells against specific antigens. This process is linked to ROS in several ways. The release of cancer antigens is regulated by the type of cell death, with autophagy or apoptotic cell death determining the release of cancer antigens. The amount of ROS present during antigen presentation affects its efficacy, with inhibition of ROS significantly reducing antigen uptake by DCs. However, modest ROS levels are required for T cell activation and differentiation. NOX2-derived ROS are involved in CD3/CD28 stimulation-mediated CD8 + T cell activation, and TCR activation promotes T cell activation by inducing ROS production and regulating IL2 and IL4 expression. Activated T cells express chemokines in response to ROS, which facilitate their migration to the tumor site, where they can induce apoptosis by expressing death ligands such as FasL and TRAIL. ROS are also involved in IL-2-dependent IL-2 production, and subsequent TNF, IFN-γ, perforin, and granzyme B production, as TCR signaling is sensitive to ROS

In 2013, some scholars proposed the concept of tumor immune circulation and elucidated the mechanism by which the immune system eliminates tumor cells. This process involves the capture, processing, and presentation of tumor-produced antigens by DCs, which initiates and activates effector T cell responses against cancer-specific antigens. Eventually, activated T cells migrate to the tumor site and eliminate target cancer cells [140]. Some links between this process and ROS have been confirmed [141]. However, excessive ROS can severely inhibit the effector function of lymphocytes, making the TME unfavorable for infiltrating lymphocytes. Evidence suggests that effector memory T cells, which have lower cytoplasmic antioxidant levels than memory T cells, can better inhibit tumor growth than memory T cells with higher intracellular GSH or other antioxidant chemicals [142]. Therefore, as we will discuss later, strict control of ROS through antioxidant mechanisms is essential for cancer immunotherapy to be effective (Fig. 4).

Cancer antigen release, presentation, priming and activation of T cells, and direct elimination of tumor cells are the four key processes of tumor immune circulation, and every single one of these procedures involves ROS. The release of cancer antigens is dependent on the type of cell death that occurs, with immunogenic or necrotic cell death stimulating antigen release and tolerogenic or apoptotic cell death inhibiting it [143]. ROS plays a role in regulating autophagy or apoptotic fate, and thus, the release of cancer antigens. Inhibition of ROS can significantly reduce antigen uptake by DCs, the most significant professional antigen-presenting cells (APCs) in vivo. Studies on tumor cell-derived microparticles (T-MPs) have shown that ROS in DCs increase lysosomal pH, promoting MHC I tumor antigen-peptide complex formation [144]. The secretion of ROS is associated with T cell activation, differentiation, and survival, with modest ROS production being necessary for T cell activation while excess ROS suppresses T cell activity. NOX2-derived ROS are involved in CD3/CD28 stimulation-mediated activation of CD8^+^ T cells. TCR activation induces ROS production and promotes T cell activation by regulating IL2 and IL4 expression. In addition, ROS can also regulate the direction of T cell differentiation [145]. By releasing perforin and granzyme and by expressing death ligands like FasL and TRAIL, activated CD8^+^ T cells directly kill tumor cells. It has been suggested that signal transmission by TCR may involve ROS or be sensitive to ROS, as TCR cross-linking induces rapid production of hydrogen peroxide and superoxide anions and stimulates activation of the FasL promoter [146]. Additionally, ROS are important for CD8 T cell effector maturation, as suppression of early mitochondria lowers the generation of IL-2, which is dependent on mROS, and the subsequent production of IL-2-dependent TNF, IFN-γ, perforin, and granzyme B. In conclusion, controlling ROS levels is crucial for the immune system to effectively destroy cancer cells, highlighting the potential of targeting ROS regulation as a therapeutic approach in cancer immunotherapy [147].

## Relationship between immunity and two regulatory cell deaths

Apoptosis is important in immune responses by promoting B-cell recruitment, the normal expansion and selection of B cells within germinal centers, and the expansion of plasma cells [148]. The redox status of the B-cell microenvironment varies significantly from the bone marrow to the periphery, and various checkpoints during cell development determine the subsequent course based on the success of each step. Emerging evidence suggests that ROS and redox reactions act at multiple levels of B-cell presence and development. Apoptosis is a key downstream response regulated by ROS. T cells are no exception to this phenomenon [149]. Death receptor-induced apoptotic signaling intermediates have significant effects on T-cell homeostasis, and DISC components such as caspases, FADD, and c-FLIP are necessary for DR-induced apoptosis and T-cell homeostasis and tolerance [150]. Moreover, cytotoxic cells such as CTL, NK, and LAK cells rely on apoptosis for their cytotoxic effects, including granule exocytosis and the death ligand/death receptor system [151]. Excessive apoptosis can lead to degenerative diseases, whereas in other cases, too little apoptosis is the cause of the problem. One of the conditions where there is insufficient apoptosis and there are persistent malignant cells is cancer. Despite being one of the causes of cancer, defective apoptosis is a key component of cancer therapy because it is a popular target for many immunotherapies. Each defect or abnormality in the apoptotic pathway may also be a target for cancer therapy. Cancer cells may be destroyed by medications or therapeutic methods that can return apoptotic signaling pathways to normal, which makes room for potential new anticancer medications [152].

Autophagy is a fundamental cell biological pathway that also plays a critical role in immunity. Firstly, autophagy can assist in eliminating pathogens. Pathogens can induce autophagy by stimulating innate immune receptors, such as Toll-like receptors, while also controlling pro-inflammatory signaling. In adaptive immunity, autophagy in dendritic cells contributes to efficient processing and presentation of MHC class I or II intracellular antigens, particularly by promoting a dying tumor cell’s release of DAMPs, which are then presented to CD8^+^ cytotoxic lymphocytes, thus eliminating tumor cells. Autophagy may also be crucial in activated T cells. Early on in cell activation, access to nutrients is constrained. Autophagy is a crucial mechanism for recycling amino acids, which is necessary to complete an efficient transition [150]. Additionally, it plays a role in subsequent further development and T-cell polarization. HSCs’ ability to renew themselves, B1 cell growth, plasma cell survival, and IgG production are all impacted by autophagy [153].

##  Current and potential ROS-related therapeutic strategies for cancer therapy

### ROS-related cancer treatment strategies and clinical trials

#### ROS and cancer chemotherapy

Chemotherapy is a commonly used clinical approach for cancer treatment, which primarily induces oxidative stress to elevate ROS levels and disrupt redox homeostasis in cancer cells. This ultimately leads to damage in tumor cells, with ROS levels exceeding a certain threshold. Research has indicated that certain chemotherapeutic agents, including doxorubicin, daunorubicin, platinum coordination complexes, alkylating agents, and arsenicals, are capable of generating high levels of ROS [154]. Moreover, novel drugs are increasingly combining chemotherapy with oxidative stress as a potential therapeutic approach against cancer, such as gemcitabine (Gem), a selenium prodrug that enhances the efficacy of chemotherapy and oxidative stress [155]. It should be mentioned, though, that ROS also play a key part in multidrug resistance (MDR), which is a major factor in cancer treatment failure. It has been demonstrated that prolonged exposure to ROS causes resistance, possibly as a result of the activation of transcription factors that are redox sensitive (NF-B, Nrf2, c-Jun, and HIF-1). These elements may then increase the expression of proteins that support cell survival [156]. For instance, NOX1-induced HIF1α specifically increases multidrug resistance gene 1 (MDR1) expression, which counteracts excess ROS and leads to cancer cells’ enhanced expression of P-gp and drug resistance [157]. Therefore, ROS inhibition can have a beneficial effect on tumor treatment.

#### ROS and cancer radiotherapy

Radiation therapy is a commonly used cancer treatment that relies on ionizing radiation (IR) to destroy cancer cells. IR can directly produce ROS through ionization, which can cause cellular damage and ultimately destroy cancer cells [158]. It is crucial to keep in mind, though, that IR-induced ROS can potentially have a second effect by encouraging the EMT, which can cause cancer cells to invade and spread. This can hinder the success of cancer treatment [159]. Radiotherapy is also closely related to the immune response, and studies have shown that combining PLGA-R837Cat based radiotherapy with clinically approved immunotherapy can produce a significant synergistic systemic treatment effect in inhibiting tumor metastasis, as well as providing long-term immune memory protection to prevent cancer recurrence [160].

#### ROS versus photodynamic therapy

Photodynamic therapy (PDT) involves the use of three parts: photosensitizer (PS), oxygen, and light. PS is applied to the tumor site and activated by specific light, leading to the production of ROS in the presence of oxygen. This mechanism triggers immune and inflammatory responses, promoting anti-tumor immunity by releasing secondary inflammatory mediators [161]. PDT can be used with immunotherapy, including immune checkpoint inhibitors and chimeric antigen receptor T-cell treatment, to create an organic combination. The main method of photoimmunotherapy is to first directly eradicate the tumor, then trigger an immune response specific to the tumor to get rid of any remaining tumor cells and metastases [162].

#### ROS and immunotherapy

The oxidative environment has a significant impact on tumor cells, immune cells, and their interactions, meaning that ROS not only play a role in conventional anticancer therapies but also in tumor immunotherapy. Due to their cytotoxicity, T cells are currently the focus of the immune system in the treatment of cancer. Adoptive cell therapy (ACT) and immune checkpoint inhibitors (ICIs) are important components of immunotherapy treating malignancies [163]. Immune checkpoint proteins prevent excessive T cell activation, thus protecting healthy tissues from immune attack. However, tumor cells can up-regulate immune checkpoint proteins, such as PD-1/PD-L1, and escape from the immune system. PD-L1 is closely associated with ROS, and its elevated membrane expression on cancer cells is associated with high HIF-1α expression. Hypoxia leads to a rapid and selective up-regulation of PD-L1 in tumors, and this up-regulation is dependent on HIF-1α, which is tightly associated with ROS. Studies have analyzed the effects of various pharmacological ROS modulators (including TrxR1 inhibitors, Nrf2 signaling modulators, free radical scavengers, etc., such as Ethaselen, Edaravone, A. formosanus extract) on PD-(L)1 expression, and explored the interaction between ROS, HIF-1α, NFκB signaling pathways, and PD-L1 expression [164]. Understanding this relationship can help design new drug combinations between anti-PD-(L)1 and ROS modulators [165]. Adoptive cell therapy using cytotoxic lymphocytes is an effective immunotherapy against tumors. However, an unfavorable tumor microenvironment with elevated levels of ROS can impair T cell function. Increasing evidence suggests that ROS influence T cell activation, proliferation, and survival, suggesting that modulation of ROS in the immune microenvironment could enhance antitumor immune responses. Pretreatment with antioxidants such as GSH and NAC significantly improved the persistence of adoptively transferred cells. Some drugs have been reported to act as strong activators of Nrf2, allowing cells to acquire high ROS resistance and can be used to enhance the efficacy of adoptive cell therapy [166]. Relevant clinical trials currently ongoing or completed are presented in Table 3 below.

**Table 3:** Current mainstream clinical trials of four ROS-related cancer treatment measures

Conditions	Interventions	Phase	Status	Identifier
**Cancer chemotherapy**				
Head and Neck Cancer	Cisplatin and XRT	Phase 2	Recruiting	NCT02994069
Bladder Cancer	Gemcitabine plus cisplatin	Not Applicable	Completed	NCT01801644
Melanoma	Elesclomol (STA-4783) and Paclitaxel	Phase 3	Terminated	NCT00522834
Breast Cancer	Paclitaxel followed by Doxorubicin	Phase 2	Completed	NCT00096291
**Cancer radiotherapy**				
Breast Cancer	Radiation therapy	Not Applicable	Completed	NCT00836186
Lung Cancer	Radiation therapy	Phase 2	Completed	NCT01055197
Primarily Resectable Pancreatic Cancer	Neoadjuvant photon radiation	Not Applicable	Completed	NCT01027221
Pancreatic Cancer	Radiation and Anti-PD-1 Antibody	Phase 2	Unknown	NCT03374293
**Photodynamic therapy**				
Non Small Cell Lung Cancer	Photodynamic Therapy	Not Applicable	Terminated	NCT03564054
Advanced Rectal Cancer	Photodynamic Therapy	Phase 2/3	Suspended	NCT01872104
Colon Cancer	PDT with 5-ALA radiosensitization	Phase1/2	Withdrawn	NCT01522677
**Immunotherapy**				
Extensive stage small-cell lung cancer (ES SCLC)	Thoracic radiotherapy and immune checkpoint inhibitors (ICI)	Phase 3	Recruiting	NCT05223647
Bladder Cancer	Anti-PD-1 (Nivolumab)	Phase 1	Terminated	NCT03106610
Colorectal Cancer Stage III	Anti-PD-1 antibody-activated TILs	Phase1/2	Unknown	NCT03904537
Esophageal Neoplasms	An anti-PD-1/PD-L1 antibody plus an angiogenesis inhibitor	Not Applicable	Recruiting	NCT05349045
Locally Advanced and Metastatic Pancreatic Cancer	PD-L1/CTLA4 BsAb	Phase1/2	Recruiting	NCT04324307
Breast Cancer	Anti PD-L1 Antibody + Anti CTLA-4 Antibody	Phase 2	Terminated	NCT03430466
Stage 0/1 Breast Cancer	IV/oral n-acetylcysteine	Phase 1	Completed	NCT01878695
Bladder Cancer	Dendritic cells	Phase 2	Completed	NCT04184232
Renal Cancer	Dendritic Cell Tumor Fusion Vaccine + Granulocyte Macrophage Colony Stimulating Factor (GM-CSF)	Phase1/2	Active, not recruiting	NCT00458536
Refractory Metastatic Colorectal Cancer	NKG2D CAR-NK	Phase 1	Recruiting	NCT05213195

*Abbreviations*: *XRT *X-Ray Therapy, *PD-1 *Programmed cell death protein 1, *ICI *Immune checkpoint inhibitors, *5-ALA *5-aminolevulinic acid, *ES SCLC *Extensive stage small-cell lung cancer, *TLR *Toll-like receptor, *PD-L1 *Programmed death-ligand 1, *CTLA-4 *Cytotoxic T-lymphocyte-associated protein 4, *BsAb *Bispecific monoclonal antibody

### ROS-related cancer treatment strategies with potential clinical effects

Most of the immunotherapies discussed above focus on enhancing T cell responses, but despite their success, only a limited number of patients benefit from these treatments. Therefore, it is urgent to explore the use of innate immune cells in the tumor microenvironment as a new approach to cancer therapy. Innate immunity comprises DCs, monocytes, macrophages, NK cells, and other cells that recognize tumors, induce and expand immune responses, promote T cell effector function, use the direct anti-tumor effector function of innate immune cells, and alleviate immunosuppression in the tumor environment [167]. Human macrophages have been engineered using chimeric antigen receptors (CARs), and due to their unique abilities to penetrate tumors, CAR-MS infusion has been shown to prolong overall survival in mouse models of solid tumor transplantation. However, this technology requires further clinical practice [168]. Dendritic cell vaccines are crucial for therapeutic cancer vaccination, as DCs transport and cross-present tumor antigens to activate cytotoxic T lymphocytes and trigger MHC class I and II immunity [169]. Nevertheless, not all tumors express immunogenic neoantigens and fail to induce effective immunity [170].

In the tumor microenvironment, CAFs form a heterogeneous group of stromal cells with varied phenotypes and functions across different cancer types. CAFs are known to promote tumorigenesis by suppressing immune responses. Studies have demonstrated that inhibition of IDO1, NOX2, and NOX4, as well as reduction of CAF-induced ROS production in MDSCs, can restore immune responses in the tumor microenvironment [171]. Therefore, ROS production-associated targets may represent potential therapeutic approaches to reverse CAF-mediated immunosuppression. Due to the potent immunomodulatory capacity of CAFs, there has been a rapid increase in the number of preclinical experiments with CAF-targeted therapies to restore antitumor immune responses. In general, there are three main strategies: CAF depletion, inhibition of CAF function, and restriction of CAF-induced ECM remodeling. However, CAF targeting faces many obstacles and challenges, particularly the lack of specific cell surface markers. To improve the effectiveness of CAF-targeted medicines, additional research is needed to find particular cell surface indicators for CAFs [172].

Noncoding RNAs (ncRNAs) play critical roles downstream of ROS in regulating physiological responses. A large number of ROS-related ncRNAs have been defined as therapeutic agents or targets for cancer, mainly including miRNAs, lncRNAs and circRNAs. Among them, microRNAs (miRNAs) are important ncRNAs that bind to mRNAs and control their degradation or translation, thereby regulating target gene expression [173]. Dysregulation of miRNAs has been causally associated with the development of many cancers, where miRNAs can act as tumor suppressors or oncogenes. Dysregulated miRNAs regulate tumor physiological processes, such as tumor cell growth or death, metastasis, and angiogenesis. There is increasing evidence of crosstalk between miRNAs and ROS, where miRNA processing is regulated by ROS that can induce or inhibit miRNA expression by regulating transcription factors or epigenetics [174]. Therefore, miRNA mimics and molecules targeting miRNAs (antimiRs) are promising therapeutic options, and some miRNA-based therapies are currently being developed. For instance, tumor suppressor miR-34 mimics from phase I clinical trials and anti-miRs against miR-122 for phase II trials have been investigated [175]. However, the details of the interactions between ROS and miRNAs remain not well understood, and their dual roles in cancer progression suggest that treatment remains subject to many limitations. Hence, specific ncRNAs should be carefully selected as therapeutic targets for different situations.

Autophagy is generally employed as a survival mechanism in tumor cells, making autophagy inhibition a novel anticancer therapeutic strategy. Studies have demonstrated that autophagy is associated with chemoresistance in tumors, involving caspase activation and prostaglandin E. Inhibition of autophagy may lead to chemosensitization, enabling synergistic action with certain anticancer drugs and overcoming drug resistance, thereby facilitating long-term treatment [176]. However, it should be noted that autophagy inhibition might not always be beneficial for tumor treatment. In some cases, inhibiting autophagy may trigger tumor signal transduction and distant metastasis. For instance, in breast cancer, autophagy inhibition leads to increased expression of ROS, subsequently promoting the expression of MIF, which may be associated with the paracrine effect of tumor signaling [177]. Similarly, in gastric cancer cells or ovarian cancer cells, autophagy inhibition has been found to promote epithelial-mesenchymal transition (EMT) and metastasis through the ROS/NF-κB/HIF-1α pathway [178, 179]. Through studies in breast cancer, researchers have discovered that autophagy plays a crucial role in preventing fibrosis within tumors. Consequently, inhibiting autophagy can activate the fibronectin integrin signaling axis, potentially leading to metastasis [180].

Given the complex role of autophagy in tumors, it is essential to enhance therapeutic efficacy, prevent possible drug resistance, and improve prognosis through innovative combination therapies. Building on the mechanisms mentioned earlier regarding metastasis and drug resistance in tumors, combining autophagy inhibitors with antioxidants or NF-κB inhibitors may prove beneficial when devising autophagy-based cancer treatment strategies. Experiments conducted with mouse melanoma cells and human ovarian cells have revealed that cells expressing high levels of PD-L1 receptor are more sensitive to autophagy inhibitors compared to cells with weak PD-L1 expression. New research is focusing on the combination of autophagy inhibitors and immune checkpoint inhibitors [181, 182]. Additionally, for glioblastoma, the combination of imipramine with drugs that inhibit VEGF signaling has shown a significant synergistic effect. This approach leads to increased autophagy levels and enhanced infiltration of glioma by T cells, thereby promoting immunity through autophagy [183].

### Rational use of dual acting ROS

Indeed, the role of ROS in cancer therapy is complex and context-dependent. Rational use of ROS with dual roles is an urgent problem to be solved. While there are many different types of ROS, they are often regarded as a whole, and there are few studies and comparisons on the role of different types of ROS in treatment. Thus, how to select ROS for therapy has not yet been clarified. Furthermore, the boundary between promotion and inhibition must be carefully considered to avoid unintended consequences.

Current mainstream research on treatment resistance includes chemoresistance and radioresistance. For chemotherapy, redox signaling is a feasible target to overcome cancer chemoresistance. However, the mechanism of acquired chemoresistance may be paradoxical, as recent studies have shown that treatment of resistant cells with drugs with antioxidant capacity can reduce intracellular ROS, and MDR inhibition may be partially attributed to ROS inhibition [184]. On the other hand, low quantities of H_2_O_2_ enhance P-gp expression in cancer cells, although greater levels of ROS may also negatively influence P-gp expression [185]. Therefore, more research is needed to pinpoint the precise molecular mechanisms regulating P-gp and other multi-drug resistant proteins via ROS. Given the complex role MRP1 plays in resistance, it is theoretically possible to directly assess mRNA levels in real time or determine Pgp1 expression levels at the protein level during medication, which can subsequently be fundamentally addressed using related protein inhibitors [186].

Radiation therapy can induce EMT and cancer stem cell (CSC) phenotype, leading to drug resistance in cancer therapy. Overcoming chemoresistance can be achieved by targeting CSC, EMT, and oncogenic metabolic pathways, which are mainly achieved by acting on a number of transcription factors and signaling pathways, including the Snail, STAT3, PI3K/Akt pathway, and MAPK cascade [159]. For example, blockade of TGF-β signaling by LY2109761, a TGFβR-I kinase inhibitor, enhances radiation response in glioblastoma [187]. Targeting the PI3K/AKT/mTOR signaling pathway can act as radiosensitization in head and neck squamous cell carcinoma [188]. Inhibitor blockade of Akt may also contribute to inhibition of IR-induced EMT and increased radiosensitivity [189]. CSC contains low ROS levels and strong ROS defense mechanisms, and BSO pretreatment of Thy1CD24L-rich CSC cells resulted in significant radiosensitization by GSH depletion [190].

In summary, the use of ROS as a therapeutic strategy requires careful consideration of the specific type of ROS, the optimal dose, and the target of the therapy. To completely understand the nuanced role of ROS in cancer therapy and to pinpoint precise ROS targets for efficient treatment, more research is required.

## Conclusions and prospect

The redox state is critical for regulating programmed cell death, including autophagy and apoptosis, as well as immune function, and acts as an intermediary of crosstalk. Oxidoreduction imbalance is then one of the most important hallmarks of tumors, and ROS are also significantly different in generation or action among different components of TME due to different signaling pathways that stimulate ROS production versus action. Cell death and immunity are both important physiological processes and have different types of regulatory mechanisms. These regulatory factors mainly include transcription factors and kinases, which can coexist in cells to show mutual promotion or inhibition. Under the combined action of different mechanisms, the regulation of ROS can generally be summarized as a dual role, that is, according to the type of ROS and the period in which the cell is located, it selectively plays a promoting or inhibiting role. It is reflected in both pro- and antitumor effects in cancer. Here we simply elaborate on the pathway of action of the dual role of ROS, but it is unknown what role it plays at what stage, for example, tumor stage specificity may affect tumor cell responses to ROS. In the context of redox reactions, there is always a significant causal relationship between cell death and immune function, whether in normal immune or pathological conditions, reflected in that ROS can determine the fate of immune cells (including innate or adaptive immune cells) by regulating autophagy or apoptosis, or even directly acting on immune system components, thereby affecting immune function. Taken together, the intricate relationship between immunity and cell death associated with redox instability plays an important function in tumor-predominant pathomechanisms, which provides possible guidance for subsequent treatment of human diseases. Interventions represented by chemoradiotherapy, photodynamic therapy, and immunotherapy have been widely used, and there are also relevant clinical (pre) trials of possible ROS-related treatments including innate immune cells, CAF, ncRNA, etc., providing a direction for further research. However, the optimal dose and regimen of ROS for cancer treatment have not yet been clearly determined, and exactly what types are used, when they increase, and when they inhibit are not clear. It is worth investigating in the future to select more effective drug combinations for each treatment and to gain a broad understanding of the cellular events occurring in each specific tumor category (tissue, cell, stage, ROS level) through a facile approach to easily induce cancer cell death and tumor regression in vivo by tightly regulating the balance between ROS accumulation and ROS-induced autophagy or apoptosis and immunity. Personalized medicine, which employs a particular treatment for a patient to get a therapeutic diagnosis, is undoubtedly the way that cancer will be treated in the future.

## Data Availability

Not applicable.
